# Feasibility of Bedside Ultrasound-Guided Peripherally Inserted Central Catheter Placement in Cancer Patients in Palliative Care: A Single-Center Retrospective Study

**DOI:** 10.3390/medicina61101876

**Published:** 2025-10-19

**Authors:** Hak Ryeong Kim, Junyong Lee, Jang Yong Kim, Hwa Sun Kim

**Affiliations:** 1 Department of Family Medicine, Veterans Health Service Medical Center, Seoul 05368, Republic of Korea; 2 Division of Vascular and Transplant Surgery, Department of Surgery, Seoul St. Mary’s Hospital, College of Medicine, The Catholic University of Korea, Seocho-gu, Seoul 06591, Republic of Korea

**Keywords:** PICC placement, terminal cancer, palliative care, quality of life

## Abstract

*Background and Objectives*: Some cancer patients in palliative care need to access intravenous administration of medications to relieve symptoms. Few studies have explicitly assessed the safety and feasibility of peripherally inserted central catheter (PICC) insertion at bedside in palliative care settings. In this study, we suggest the usefulness, safety, and feasibility of bedside ultrasound-guided PICC placement as a tool for improvement in the quality of life for patients in palliative and hospice care settings. *Materials and Methods*: The study population, with terminal cancer and admitted to a palliative and hospice care unit in the Veterans Health Service Medical Center, was evaluated (N = 150). The patients were divided into two groups based on the methods of PICC insertion: Group 1 (PICC at bedside, N = 75) and Group 2 (PICC in intervention room, N = 75). The two groups were matched for age, sex, the level of Eastern Cooperative Oncology Group (ECOG) performance status, and types of primary cancer. *Results*: The success rates of the PICC procedure for Groups 1 and 2 were 89.33% and 97.33%, respectively, with no significant difference between the groups (*p* = 0.102). The mean duration (days) of catheter use was longer in Group 1 (23.31 ± 16.36) compared to that in Group 2 (21.90 ± 18.95), with no statistically significant difference (*p* = 0.639). Multivariable logistic regression analyses confirmed that Group 1 was not inferior to Group 2 regarding procedural success (Model 2, *p* = 0.21) and catheter dwell time (Model 2, *p* = 0.66). The most common cause of catheter removal in both groups was death, followed by self-removal and hospital discharge (*p* = 0.386). *Conclusions*: This study suggests that ultrasound-guided PICC insertion at bedside may have comparable procedural outcomes with potentially reducing the risks associated with intra-hospital patient transport compared with fluoroscopy-guided placement. We suggest this bedside approach can be considered a feasible and safe method for improving the quality of life of patients in palliative care settings.

## 1. Introduction

According to the World Health Organization (WHO) and GLOBOCAN 2022, global cancer incidence reached approximately 19.97 million in 2020, reflecting an increase of about one million cases (10.3%) compared with that in 2018 [1,2,3,4,5]. Additionally, the Worldwide Hospice and Palliative Care Alliance estimated that, as of 2017, approximately 25.73 million people worldwide required palliative care, with 28% of them having terminal cancer [6]. In 2021, a total of 1.72 million patients in the United States were registered with the National Hospice and Palliative Care Organization, with cancer patients accounting for 23% (approximately 395,600 patients) [7]. Similarly, in South Korea, the increasing prevalence of cancer has led to increased palliative and hospice service utilization. According to the Korean Statistical Information Service from Statistic Korea, the national cancer prevalence rate increased by 195% from 950.5 per 100,000 in 2007 to 1859.5 per 100,000 in 2021 [8]. Further, the Ministry of Health and Welfare and National Hospice Center reported that in 2022, the palliative and hospice service utilization rate among cancer-related deaths was 24.2% [9].

As patients near the end of life, they often experience distress symptoms such as cancer pain, dyspnea, delirium, and death rattle. In this stage, administering medications or providing nutritional support is often considered futile [10]. However, a number of patients receiving palliative and hospice care experience a variety of symptoms such as generalized weakness, chronic pain, lethargy, appetite loss, cachexia, progressive dysphagia, and an altered level of consciousness, which often require appropriate medical and nutritional support [11,12]. In such cases, symptom control can be achieved through subcutaneous (SC), intramuscular (IM), or intravenous (IV) injections. However, SC or IM injections often cause pain, leading to inconsistent results, and further obtaining IV access could be challenging due to the poor vascular patency of peripheral vessels resulting from previous cancer treatment such as radiation therapy or chemotherapy [13,14,15]. Various central venous access methods are available, such as chemoports, subclavian and femoral central venous catheters (CVCs), and PICCs. PICCs have emerged as a plausible method for patients receiving palliative and hospice care [13,15]. Compared with PICCs, CVCs have a shorter lifespan and carry a higher risk of complications (e.g., pneumothorax, infection, and central venous injury) due to their proximity to central veins. While chemoports as CVCs are used for regular chemotherapy administration, the procedure of chemoport placement, which is performed subcutaneously through skin incision, is associated with higher complication risks, and chemoport removal is more difficult in the case of infection compared to PICC removal as well [15,16].

A PICC is a type of central venous catheter inserted through peripheral veins in either upper arm [14]. Once in place, a PICC can be used for several weeks to months, depending on maintenance quality, and serves purposes such as administrating antibiotics, total parenteral nutrition (TPN), and blood sampling [17,18,19]. PICC insertion can be performed either under fluoroscopic guidance in the interventional radiology (IR) room or at the bedside using ultrasound guidance. Most patients with terminal cancer receiving palliative care are older adults with at least one chronic comorbidity, and they often experience controllable symptoms such as breakthrough pain, fatigue, dyspnea, anxiety, and depression [20,21]. Transporting these patients to an IR suite for fluoroscopic-guided PICC insertion may compromise their comfort and quality of life by exacerbating dyspnea, increasing procedural anxiety, inducing stress, and causing adverse reactions to contrast agents. In contrast, PICC placement performed at the bedside via ultrasonography could provide a stable and comfortable environment with emotional support, making it a more appropriate access for patients in palliative and hospice care settings who require vascular access. According to a prospective observational study by Kim HJ et al., which compared a subclavian venous catheter (SVC), chemoport, and PICC in cancer patients, the chemoport was found to be more significantly suitable for long-term use, such as for chemotherapy. Additionally, the PICC had an extended usage period compared to the SVC [16]. In terminal cancer patients, routine PICC placement has been shown to significantly reduce discomfort and provide greater comfort compared to peripheral IV access [14]. Although some discomfort was reported during the PICC insertion procedure, 83% of patients reported positive satisfaction afterward [22]. Despite the growing use of PICCs in various clinical settings, few studies have specifically evaluated the safety, efficacy, and utility of bedside PICC insertion in palliative care environments. According to the European Society of Anaesthesiology (ESA) guidelines, the choice between PICCs and chemoports has specific advantages depending on the clinical context. Chemoports are more suitable for long-term chemotherapy, whereas PICCs are less invasive and easier to insert and remove [23]. In palliative care settings, where the expected duration of use is often limited and patient comfort is a priority, PICCs may represent a practical option.

This study aims to explore the potential of bedside ultrasound-guided PICC placement in terms of enhancing the quality of life of patients receiving palliative and hospice care by providing more timely accessible vascular access, particularly for those with advanced cancer in palliative and hospice care settings.

## 2. Methods

### 2.1. Study Design and Patients

This retrospective study was conducted on patients admitted to the palliative and hospice care ward at the Veterans Health Service (VHS) Medical Center from October 2022 to September 2024. This study was approved by the Institutional Review Board Committee of the VHS Medical Center (IRB No. 2024-10-003).

During the study period from October 2022 and September 2024, a total of 297 patients who underwent peripherally inserted central catheter (PICC) placement for palliative care were identified from our institution’s electronic medical records using procedural codes. Ultrasound-guided PICC placements performed at the bedside were identified using the code “sono-guided PICC puncture,” typically applied to critically ill or non-transportable patients. Fluoroscopy-guided placements conducted in the interventional radiology (IR) suite were identified using the code “Central Venous Catheter/Peripherally Inserted with Fluoroscopy,” generally used for clinically stable patients who could be safely transported. No additional clinical exclusion criteria (e.g., active infection, coagulopathy, or thrombocytopenia) were applied in this step. The patients were divided into two groups according to the method of insertion: Group 1 underwent ultrasound-guided PICC insertion at the bedside, while Group 2 underwent the procedure in the IR suite under fluoroscopic guidance. To ensure comparability between the two groups, we applied exact matching using R software (version 4.1.2; R Core Team, Vienna, Austria), based on sex, ECOG performance status, and the type of terminal cancer diagnosed. Age was categorized into four groups, 60–69, 70–79, 80–89, and ≥90 years, and matched accordingly. For each group, patients with identical values for these covariates and within the same age category were identified. When multiple potential matches existed, random selection was performed within R to minimize biased pairing. Patients who could not be matched under these criteria or were not selected during the random paring step were excluded. After this matching procedure, 75 patients remained in each group, resulting in a total of 150 patients included in the final analysis.

The types of terminal cancer diagnoses were categorized into head and neck, lung, gastrointestinal, genitourinary, and hematologic cancers. Head and neck cancers included oral, tongue, and maxillary cancers and meningioma. Gastrointestinal cancers encompassed esophageal, gastric, colorectal, liver, pancreatic, gallbladder, bile duct, and appendix cancers. Genitourinary cancers included prostate, bladder, cervical, kidney, and ovarian cancers. Hematologic cancers were represented by lymphoma. Comorbidities were classified into three main categories: cerebrovascular/cardiovascular diseases (including hypertension, coronary artery disease, and cerebrovascular disease), respiratory diseases (including chronic obstructive pulmonary disease and asthma), and metabolic or renal diseases (including diabetes, chronic kidney disease, and dyslipidemia).

### 2.2. PICC Insertion

In Group 1, a 5-French (Fr.) dual-lumen Power PICC (Bard Access System, Salt Lake City, UT, USA) was used, and the procedure was performed under sterile conditions. Ultrasonography was used to identify suitable veins with no contraindications in both upper arms. The insertion site was disinfected with 2% chlorhexidine, and the practitioner wore a sterile gown and used a sterile drape. Local anesthesia was administered with 2% anhydrous lidocaine HCl. Using a modified Seldinger technique, the vein was punctured with a micro-puncture needle under ultrasound guidance. Then, a guidewire was inserted, followed by peel-away sheath placement into the vein. The PICC length was measured from the insertion site to the axilla, from the axilla to the sternum, and from the sternum to the third intercostal space. Next, the catheter was trimmed accordingly. After insertion, the position of the catheter tip was verified via ultrasonography to ensure that it had not entered the internal jugular vein. In addition, ultrasonography was used to track the catheter from the subclavian to the initial portion of brachiocephalic vein to confirm that the tip was advancing properly. As an additional confirmation, the catheter was flushed with saline to check for turbulence in the internal jugular vein before the sheath was removed. Post-procedure chest radiography was performed to verify the position of the catheter tip.

In Group 2, the same 5-Fr. Dual-lumen Power PICC was used. Under fluoroscopic guidance, a guidewire was advanced to the superior vena cava (SVC), and the PICC length was measured as aforementioned. Real-time fluoroscopy was used to monitor the catheter insertion and confirm the position of the catheter tip. All other procedural steps were identical to those performed in Group 1.

### 2.3. Variable Definitions and Outcomes

Successful PICC placement was defined as catheter insertion into the SVC, whereas malposition was defined as insertion into the ipsilateral internal jugular vein or other vessels. The reasons for failure were categorized as puncture failure due to inability to locate a suitable vein or failure to insert the guide wire after venipuncture, malposition where the catheter tip was not positioned in the SVC post-procedure, and inability to maintain patient positioning during the procedure due to delirium or pain in other areas.

The terminal cancer diagnosis was based on the primary cancer diagnosis, and comorbidities were defined according to the patients’ electronic medical records. Laboratory test results and anticoagulant use were evaluated on the basis of data recorded at the time of the PICC insertion order. Catheter duration was calculated from the day of insertion to the day of removal, and the reasons for catheter removal were classified as death, discharge, or self-removal.

### 2.4. Statistical Analysis

Continuous variables were analyzed using the two-sample *t*-test, and categorical variables were analyzed using the chi-square test. Multivariable logistic regression analysis was performed to investigate factors associated with the success of the procedure. The unadjusted model included only the procedure setting (Group 1, ultrasound guidance at bedside, versus Group 2, fluoroscopic guidance in IR). Model 1 was adjusted for sex and age. Model 2 further included ECOG performance status and primary cancer diagnosis. In each model, odds ratios (ORs) with 95% confidence intervals (CIs) were calculated. Statistical analysis was performed using Rex software (version 3.6.3; Rex Soft Inc., Seoul, Republic of Korea) and R (version 4.1.2; R Core Team, Vienna, Austria), and a *p*-value of <0.05 was considered statistically significant.

## 3. Results

### 3.1. Baseline Characteristics

**Figure 1:**
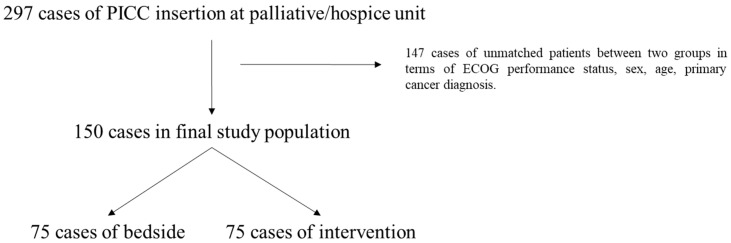
Schematic study flow. PICC, peripherally inserted central catheter; ECOG, Eastern Cooperative Oncology Group.

Out of the 297 patients who underwent PICC insertion, 150 patients (75 in each group) were included in this study (Figure 1). The general characteristics of the study population are shown in Table 1. There were no significant differences in key demographic variables between the two groups. The average height, weight, and BMI were similar between the two groups, with no significant difference (*p* > 0.05). The majority of participants were male (77.33% in both groups), and there was no significant difference in sex distribution (*p* > 0.99). Age distribution was also comparable, with no significant difference between groups (*p* > 0.99). The age distribution was as follows in both groups, with the highest proportion of patients in the 70–79 years range: 60–69 years, 6.67%; 70–79 years, 42.67%; 80–89 years, 33.33%; and 90–99 years, 17.33%. The primary cancer type diagnosed was evaluated across both groups. The most common cancer types were gastrointestinal and hepatobiliary (GI/HBP) cancers (N = 86, 57.33%), and lung cancer was the most single cancer (N = 30, 20.00%). There were no significant differences in cancer type between the groups (*p* > 0.99). ECOG performance status was also similarly distributed across both groups. Most patients had an ECOG score of 3 (54.67%), reflecting relatively fair performance status but no significant differences in ECOG performance status between the groups (*p* > 0.99). In terms of comorbidities, cerebral/cardiovascular diseases were the most common in both groups, affecting 61.33% of Group 1 and 72% of Group 2, with no significant difference between groups (*p* = 0.225) (Table 1). These characteristics demonstrate that the study groups were well-matched, and any differences in outcomes could be ascribed to the procedure rather than patients’ characteristics.

### 3.2. Laboratory and Anticoagulation Characteristics

The clinical findings of the study population are presented in Table 2. Laboratory values showed no significant differences between the two groups. Regarding procedure-related bleeding tendency, the mean hemoglobin level was 9.94 ± 2.11 g/dL, with Group 1 having a slightly higher value (10.22 ± 2.10) compared to Group 2 (9.65 ± 2.10), but this difference was not statistically significant (*p* = 0.095). Platelet counts were also comparable between groups (Group 1: 236.79 ± 131.27 × 10^3^/µL; Group 2: 239.05 ± 113.40 × 10^3^/µL; *p* = 0.910). INR as prothrombin time (PT) and activated partial thromboplastin time (aPTT) did not differ significantly between the groups (INR: Group 1: 1.15 ± 0.14; Group 2: 1.16 ± 0.17; *p* = 0.698; aPTT: Group 1: 28.85 ± 4.06 s; Group 2: 29.88 ± 7.49 s; *p* = 0.297). The majority of patients in both groups were not on anticoagulation therapy (Group 1: 82.67%; Group 2: 90.54%). A small proportion of patients used one anticoagulant (Group 1: 14.67%; Group 2: 8.11%), and an even smaller proportion used two or more anticoagulants (Group 1: 2.67%; Group 2: 1.35%). The Michigan class risk score, used to predict the risk of PICC-related deep vein thrombosis, showed a similar distribution across the groups, with most patients classified as Class III (Group 1: 58.67%; Group 2: 65.33%) and a smaller proportion as Class IV (Group 1: 41.33%; Group 2: 34.67%), with no significance between the two groups (*p* = 0.501) (Table 2) [24].

### 3.3. Procedural Outcomes

Details of the PICC procedural outcomes are shown in Table 3. The overall success rate was 93.33%, with Group 1 having a slightly lower success rate (89.33%) compared to Group 2 (97.33%), but the difference was not statistically significant (*p* = 0.102). The right upper arm was the most commonly used site in both groups (Group 1: 89.55%; Group 2: 90.41%), with no significant difference between groups (>0.99). The basilic vein was the preferred vessel for puncture (Group 1, 58.21%; Group 2, 56.16%), with no significant difference between the groups (*p* = 0.942). The initial catheter tip position was optimal in all cases in Group 2; however, 97.10% of patients had an optimal catheter position, with 2.9% classified as malposition (*p* = 0.243), in Group 1 (Table 3). The mean catheter use duration was 22.58 ± 17.71 days, with Group 1 having a slightly longer mean duration (23.31 ± 16.36 days) compared to Group 2 (21.90 ± 18.95 days), but this difference was not statistically significant (*p* = 0.639). When categorized with a 22-day threshold, 56.72% of Group 1 patients had a mean overall PICC duration of less than 22 days, while 63.01% of Group 2 patients had a shorter PICC use duration (*p* = 0.198). The primary reason for catheter removal in both groups was death (Group 1, 65.67%; Group 2, 73.97%), followed by self-removal and discharge, with no statistically significant differences between the groups (*p* = 0.386) (Table 3).

### 3.4. Multivariable Logistic Regression for Procedural Outcomes

To access factors related to procedural success, multivariable logistic regression analysis was performed (Table 4). Compared with Group 2, Group 1 showed a lower tendency toward procedural success in the unadjusted model, but the difference was not statistically significant (OR, 4.36; 95% CI, 0.89–20.0; *p* = 0.07). The adjusted model for age and sex (Model 1) and Model 2, which further included ECOG performance and primary cancer diagnosis in addition to age and sex, were also not statistically significant (Model 1: OR, 4.77; 95% CI, 0.94–25.0; *p* = 0.06; Model 2: OR, 4.28; 95% CI, 0.44–50.0; *p* = 0.21). Similarly, multivariable logistic regression for a catheter dwelling time ≥22 days showed no significant difference between the two groups (Table 5).

## 4. Discussion

The present study demonstrated the comparison of outcomes of PICC placement in different environments, at the bedside using ultrasonography or in the IR room with fluoroscopy, for patients with terminal cancer receiving palliative and hospice care. Patients in both groups were well-matched for the types of primary cancer diagnosed, sex, age, and ECOG performance levels, so comparable condition levels between the groups were ensured. There were no significant differences between the groups in terms of comorbidities, laboratory values, or anticoagulant use, indicating a high degree of similarity in disease severity and characteristics. This study minimized selection bias, enhancing the validity of study outcomes by matching the study population.

Both placement methods demonstrated high procedural success rates, with no statistically significant differences between the two groups. While Group 1 had a longer catheter duration than Group 2, this difference was not statistically significant. Additionally, catheter tip malposition occurred in only two cases in Group 1 (ipsilateral internal jugular vein and lateral thoracic vein), and there was no significant difference in verification of tip positioning between the groups. Group 1 was evaluated with post-procedure portable chest radiography, and Group 2 was assessed with real-time chest radiography. These findings indicate that bedside ultrasound-guided PICC insertion is not statistically significantly different compared with fluoroscopy-guided insertion.

Multivariable logistic regression analysis showed tendencies similar to those in the matched analysis. Procedural success in Group 1 was slightly lower than that in Group 2, but the difference was not statistically significant, after adjusting for demographic and clinical covariates. Likewise, the result for dwell time (≥22 days) also showed no significant difference. These findings suggest that the procedural success rate and catheter dwell time of bedside ultrasound-guided PICC insertion may be equivalent to those of fluoroscopy-guided insertion in intervention room for palliative care patients, with the added advantage of avoiding the risks associated with intra-hospital transport.

The risks and complications associated with intra-hospital transport for procedures and imaging have been extensively documented, with greater risk involved in critically ill patients [25,26,27,28]. Terminal cancer patients often have multiple comorbidities and advanced age (in the 70s and 80s), contributing to the burden of transport. In this context, bedside PICC insertion may substantially reduce transport-related risks.

Early initiation of palliative care in patients with terminal cancer results in substantial reductions in healthcare costs. Research has indicated considerable cost savings depending on the cancer type, mode of palliative care delivery (home-based, consultation, or inpatient), percentage of health insurance reimbursement for palliative care, and timing and duration of palliative interventions [29].

A minor limitation of bedside PICC insertion is the inability to verify catheter tip placement in real time, potentially increasing the risk of misplacement. Post-procedural chest radiographs are typically required to confirm tip placement right after the bedside PICC procedure is performed, which makes immediate correction of the catheter tip hard. Furthermore, unlike fluoroscopy, ultrasonography alone may not reveal anatomical variations in veins or central venous stenosis, potentially leading to insertion failures. ESA guidelines identify fluoroscopy as the gold standard for tip confirmation and navigation but also acknowledge the role of echocardiography-based and ECG-based navigation systems where available [23]. In Korea, ECG-based confirmation was not available during the study period, but chest radiography was used as a practical alternative for post-procedural verification. Importantly, this study incorporated additional ultrasound-based tip navigation such as exclusion of internal jugular vein malposition and tracking from the subclavian to brachiocephalic vein, which may have reduced the risk of misplacement despite the lack of real-time ECG. To address these challenges, portable intracavitary electrocardiogram (EKG)-guided PICC placement (Sherlock) has been introduced in some countries [30,31,32]. This technique enables real-time confirmation of catheter tip placement by modifying the EKG lead when the catheter tip reaches the SVC, allowing for immediate correction during the procedure and substantial improvement of procedural safety and accuracy [30,31,32].

This study has a few limitations. First, the sample size was small, with only 150 patients across the matched groups, which limits the generalizability of the findings. In addition, no sample size or power calculation was performed before the study began, as the number of patients was determined retrospectively, which may have further limited the ability to detect small but clinically significant differences. Subsequently, the matching process reduced the final sample size, which may have limited the ability to detect small differences between groups. However, a key strength of this study was the use of exact matching based on clinically relevant variables, which helped reduce potential confounding and improve the comparability between groups. Furthermore, the study reflects clinical insights in a palliative care setting and provides valuable insights into the use of PICC in this population. Given the characteristics of particular patients in palliative settings that are hard to study, the study population may not be small. Second, the wide confidence intervals in the logistic regression results suggest imprecision in some of the effect estimates, due to the limited sample size following the matching analysis and the retrospective nature of the study. This limitation should be considered when interpreting the findings.

Third, practitioners had varying levels of proficiencies. Different practitioners performed the procedures in Groups 1 and 2, owing to the differing settings for each procedure. Thus, such differences in proficiencies could not be fully accounted for, making it difficult to assess the potential bias. Third, we did not evaluate complications, e.g., catheter-related bloodstream infections or PICC-induced thrombosis. Although these results were included in the sub-data, they were excluded from the main analysis while matching the two groups, owing to the small sample size. Hence, follow-up studies with additional patient groups are needed to compare potential complications.

The goal of palliative and hospice care is to provide comprehensive holistic support to patients transitioning to the last moments of their lives. Patients with terminal cancer endure the progressive distress of approaching death, and their quality of life can be enhanced not only by physical care but also by mental, psychological, social, and spiritual care [33]. Although the necessity of intravenous access for nutritional and pharmacological support for patients in palliative settings is controversial, this approach may contribute to improving their overall quality of life. If a patient is dehydrated or requires immediate medical approaches to manage symptoms such as acute breakthrough pain or delirium, placement of a PICC may be necessary to provide continuous access.

In conclusion, bedside ultrasound-guided PICC insertion showed no statistically significant differences compared with fluoroscopy-guided placement, while potentially reducing the risks associated with patient transport or contrast use. These findings provide insights, and further prospective studies with larger sample sizes are required. Nonetheless, bedside PICC placement via ultrasonography may be a safe and feasible alternative in palliative and hospice care settings.

## Figures and Tables

**Table 1:** General characteristics of the study population.

		Total (N = 150)		Group 1 (N = 75)		Group 2 (N = 75)		*p*-Value
Demographics	Height, cm	162.97	±7.77	163.47	±7.48	162.48	±8.07	0.439
	Weight, kg	55.28	±11.19	56.34	±11.63	54.23	±10.71	0.249
	BMI, kg/m^2^	20.76	±3.68	21.01	±3.74	20.5	±3.63	0.402
Sex	Male	116	(77.33)	58	(77.33)	58	(77.33)	
Age, year	60–69	10	(6.67)	5	(6.67)	5	(6.67)	
	70–79	64	(42.67)	32	(42.67)	32	(42.67)	
	80–89	50	(33.33)	25	(33.33)	25	(33.33)	
	90≤	26	(17.33)	13	(17.33)	13	(17.33)	
Type of primary cancer	Head and Neck	16	(10.67)	8	(10.67)	8	(10.67)	
	Lung	30	(20.00)	15	(20.00)	15	(20.00)	
	GI/HBP	86	(57.33)	43	(57.33)	43	(57.33)	
	GU	16	(10.67)	8	(10.67)	8	(10.67)	
	Hematologic	2	(1.33)	1	(1.33)	1	(1.33)	
ECOG Performance status	2	14	(9.33)	7	(9.33)	7	(9.33)	
	3	82	(54.67)	41	(54.67)	41	(54.67)	
	4	54	(36.00)	27	(36.00)	27	(36.00)	
Comorbidity	Cerebral /Cardiovascular	100	(66.67)	46	(61.33)	54	(72.00)	0.225
	* Respiratory Diseases	17	(11.33)	10	(13.33)	7	(9.33)	0.607
	** Metabolic or Renal Diseases	73	(48.67)	36	(48.00)	37	(49.33)	>0.99

Data are presented as numbers (%). Group 1, patients who underwent a bedside PICC procedure; Group 2, patients who underwent a PICC procedure guided by fluoroscopic contrast in the intervention room. The study populations in both groups were matched for primary malignancy type, age, sex, underlying comorbidities, general characteristics, and ECOG performance status. Malignancies are categorized as follows: head and neck includes meningioma and oral cavity and tonsil cancers; GI/HBP includes esophageal, stomach, appendix, colorectal/liver, pancreas, and biliary malignancy cancers; GU includes prostate, bladder, ovary, and cervix malignancy cancers; hematologic malignancy includes only lymphoma; and cerebral/cardiovascular diseases include hypertension, coronary artery disease, heart failure, and cerebrovascular accidents. * Respiratory diseases refer to asthma or chronic obstructive pulmonary disease, and ** metabolic or renal diseases include diabetes mellitus, chronic kidney disease, and dyslipidemia. Abbreviations: GI, gastrointestinal; HBP, hepatobiliary and pancreas; GU, genitourinary; ECOG, Eastern Cooperative Oncology Group; PICC, peripherally inserted central catheter. These data were analyzed using the chi-square test for categorical variables, with statistical significance defined as a *p*-value < 0.05.

**Table 2:** Clinical findings in the study population.

	Total (N = 150)		Group 1 (N = 75)		Group 2 (N = 75)		*p*-Value
Laboratory value							
Hb (g/dL)	9.94	±2.11	10.22	±2.10	9.65	±2.10	0.095
Platelet (×10^3^/μL)	237.92	±122.25	236.79	±131.27	239.05	±113.40	0.910
PT (INR)	1.15	±0.15	1.15	±0.14	1.16	±0.17	0.698
aPPT (s)	29.37	±6.02	28.85	±4.06	29.88	±7.49	0.297
BUN (mg/dL)	25.05	±16.48	23.69	±14.54	26.42	±18.20	0.309
Creatinine (mg/dL)	1.01	±0.90	0.87	±0.60	1.14	±1.11	0.072
eGFR (mL/min/1.73 m^2^)	75.21	±22.84	77.8	±20.33	72.61	±24.97	0.165
Albumin (g/dL)	2.82	±0.56	2.91	±0.54	2.74	±0.57	0.066
Anticoagulation use							
None	129	(86.58)	62	(82.67)	67	(90.54)	0.370
1	17	(11.41)	11	(14.67)	6	(8.11)	
≥2	3	(2.01)	2	(2.67)	1	(1.35)	
Michigan class risk score							
Class III	93	(62.00)	44	(58.67)	49	(65.33)	0.501
Class IV	57	(38.00)	31	(41.33)	26	(34.67)	

Data are presented as the mean ± standard deviation or a number (%). Group 1, patients who underwent a bedside PICC procedure; group 2, patients who underwent a PICC procedure guided by fluoroscopic contrast in the intervention room. The study populations in both groups were matched for primary malignancy type, age, sex, underlying comorbidities, general characteristics, and ECOG performance status. Abbreviations: Hb, hemoglobin; PT, prothrombin time; aPTT, activated partial thromboplastin time; eGFR, estimated glomerular filtration rate; PICC, peripherally inserted central catheter. Statistical analyses were performed using the two-sample *t*-test for continuous variables and the chi-square test for categorical variables. Statistical significance was set at a *p*-value < 0.05. The Michigan class risk score for PICC-related thrombosis is a tool designed to predict the risk of deep vein thrombosis (DVT) in patients with a PICC. Class III (score 3–4) and Class IV (score > 4) are classified as high-risk categories.

**Table 3:** Comparisons of procedural outcomes between the groups.

Variable	Total (N = 150)		Group 1 (N = 75)		Group 2 (N = 75)		*p*-Value
Successful cases ^a^	140	(93.33)	67	(89.33)	73	(97.33)	0.102
Failed cases ^b^	10	(6.67)	8	(10.67)	2	(2.67)	
Puncture site, arm							
Right upper	126	(90)	60	(89.55)	66	(90.41)	>0.99
Left upper	14	(10)	7	(10.45)	7	(9.59)	
Punctured vessel							
Basilic vein	80	(57.14)	39	(58.21)	41	(56.16)	0.942
Brachial vein	60	(42.86)	28	(41.79)	32	(43.84)	
Initial tip location							
Optimal ^a^	140	(98.59)	67	(97.10)	73	(100)	0.243
Malposition ^c^	2	(1.41)	2	(2.90)	N/A		
Duration of use, days (N = 140)	22.58	±17.71	23.31	±16.36	21.90	±18.95	0.639
<22	84	(60.00)	38	(56.72)	46	(63.01)	0.198
≥22	56	(40.00)	29	(43.28)	27	(36.99)	
Cause of removal (N = 140)							
Death	98	(70.00)	44	(65.67)	54	(73.97)	0.386
Discharge	23	(16.43)	14	(20.90)	9	(12.33)	
Self-removal	19	(13.57)	9	(13.43)	10	(13.70)	

Data are presented as the mean ± standard deviation or a number (%). Group 1, patients who underwent a bedside PICC procedure; group 2, patients who underwent a PICC procedure guided by fluoroscopic contrast in the intervention room. The study populations in both groups were matched for primary malignancy type, age, sex, underlying comorbidities, general characteristics, and ECOG performance status. Abbreviation: N/A, not applicable. ^a^ Successful cases, optimal catheter tip placement between the superior vena cava and mid-portion of the right atrium. ^b^ Failed cases, malposition, puncture failure, and inability to maintain posture. ^c^ Location of the catheter tip in the wrong vessels such as lateral thoracic vein and ipsilateral internal jugular vein. Statistical analyses were conducted using the two-sample *t*-test for continuous variables and the chi-square test for categorical variables. Statistical significance was defined as a *p*-value < 0.05. There were no significant differences between the groups in terms of the cause of removal (death, discharge, or self-removal) or the cause of failure (puncture failure, no poisoning, or malposition). In the unmatched data, a small number of complications such as catheter-related blood stream infection (CRBSI), insertion site discomfort, and thrombophlebitis were identified; however, these patients were excluded during the final matching. And occasional single-lumen dysfunctions were observed, but as dual-lumen catheters were predominantly used in our institution, no cases of complete catheter failure were observed in either group.

**Table 4:** Multivariable logistic regression analysis for procedural success.

Variable	Odds Ratio	95% CI	*p*-Value
Unadjusted model			
Group 1	1 (reference)		
Group 2	4.36	0.89–20.0	0.07
Model 1			
Group 1	1 (reference)		
Group 2	4.77	0.94–25.0	0.06
Model 2			
Group 1	1 (reference)		
Group 2	4.28	0.44–50.0	0.21

These data are the results of using multivariable logistic regression models. Abbreviations: CI, confidence interval. Group 1, patients who underwent a bedside PICC procedure; group 2, patients who underwent a PICC procedure guided by fluoroscopic contrast in the intervention room. Model 1 was adjusted for age and sex. Model 2 further included ECOG performance status and primary cancer diagnosis in addition to age and sex. Statistical significance was defined as a *p*-value < 0.05.

**Table 5:** Multivariable logistic regression analysis for catheter dwell time (≥22 days).

Variable	Odds Ratio	95% CI	*p*-Value
Unadjusted model			
Group 1	1 (reference)		
Group 2	0.77	0.39–1.51	0.45
Model 1			
Group 1	1 (reference)		
Group 2	0.79	0.38–1.63	0.52
Model 2			
Group 1	1 (reference)		
Group 2	0.80	0.37–1.72	0.66

These data are the results of using multivariable logistic regression models for catheter dwell time. Abbreviations: CI, confidence interval. Group 1, patients who underwent a bedside PICC procedure; group 2, patients who underwent a PICC procedure guided by fluoroscopic contrast in the intervention room. Model 1 was adjusted for age and sex. Model 2 further included ECOG performance status and primary cancer diagnosis in addition to age and sex. Statistical significance was defined as a *p*-value < 0.05.

## Data Availability

The datasets used in this analysis were obtained from medical records under approval of the IRB Committee of the VHS Medical Center.

## Abbreviations

PICC	Peripherally Inserted Central Catheter
CVC	Central Venous Catheter
SC	Subcutaneous
IM	Intramuscular
IV	Intravenous
TPN	Total Parenteral Nutrition
IR	Interventional Radiology
ECOG	Eastern Cooperative Oncology Group
SVC	Superior Vena Cava
IJV	Internal Jugular Vein
ECG/EKG	Electrocardiogram
ICU	Intensive Care Unit
BMI	Body Mass Index
PT (INR)	Prothrombin Time (International Normalized Ratio)
aPTT	Activated Partial Thromboplastin Time
BUN	Blood Urea Nitrogen
eGFR	Estimated Glomerular Filtration Rate
